# Supplementary material for: Complexome profiling of the Chlamydomonas psb28 mutant reveals TEF5 as an early PSII assembly factor
Source: Plant Cell. 2025 Mar 17;37(6):koaf055. doi: 10.1093/plcell/koaf055 (PMC12164590; doi:10.1093/plcell/koaf055)
Supplement: koaf055_Supplementary_Data [file koaf055_supplementary_data.zip › psb28_interactive.zip!/plots/intensity/qualityPlots/peptides/psbF_Cre-1.g2716994.t1.p1.html]

### Cre-1.g2716994.t1.p1

Trivial name: psbF  
SeqCoverage [median]: 31.80  
Mass: 4.98 kDa  
  
 

| Sequence | MissedCleavage | Mass [Da] | wtIntensity | muIntensity | Score |
| --- | --- | --- | --- | --- | --- |
| KSAEVLVYPIFTVR | 1 | 1620.92 | 37412.18 | 0.00 | 101.36 |
| SAEVLVYPIFTVR | 0 | 1492.83 | 180771.82 | 22362.51 | 209.85 |
|  |  |  | 218184.0 | 22362.5 |  |
